# Dentin Sialophosphoprotein Expression Profile in Developing Human Primary Teeth: An Experimental Study

**DOI:** 10.30476/dentjods.2024.100219.2202

**Published:** 2025-03-01

**Authors:** Soussan Irani, Shohreh Alimohammadi, Fatemeh Ataei

**Affiliations:** 1 Dept. of Oral Pathology, Dental Research Centre, Dental Faculty, Hamadan University of Medical Sciences, Hamadan, Iran. Lecturer at Griffith University, Gold Coast, Australia; 2 Gynecologist and Perinatologist, Hamadan University of Medical Sciences, Hamadan, Iran; 3 Postgraduate Student of Prosthodontics, Dental Faculty, Hamadan University of Medical Sciences, Hamadan, Iran

**Keywords:** Dental pulp, Dentin sialophosphoprotein, Enamel organ, Odontogenesis

## Abstract

**Statement of the Problem:**

Tooth development is initiated by localized thickenings of the primary epithelial bands. Dentin sialophosphoprotein (DSPP) is the most abundant non-collagenous protein in the dentin matrix. DSPP's expression extends to multiple tissues, including dentin, cementum, and bone. However, the role of DSPP is not yet clear in the development of human tooth.

**Purpose:**

The current study aimed to examine the DSPP expression profile during the development of human primary tooth structures with a sufficiently large sample size.

**Materials and Method:**

In this experimental study, a total of 33 samples of aborted human fetuses were divided into three age groups <16 weeks, <19 weeks and ≥19 weeks. Immunohistochemistry (IHC) was performed with Anti-DSPP rabbit polyclonal antibody. A two-way ANOVA analysis was conducted to examine the differences between the groups (*p*< 0.05).

**Results:**

An increase in DSPP expression was observed with the progression of gestational weeks in different histological structures of developing human primary teeth.

**Conclusion:**

An increase in the expression level of DSPP with the progression of gestational weeks may confirm that reciprocal interactions between the enamel organ (EO) and dental pulp cells contribute to tooth formation.

## Introduction

Tooth development is initiated by localized thickenings of the primary epithelial bands. Then, tooth formation continues through three stages: bud, cap, and bell. During the late bell stage, four distinct layers can be seen in the coronal part of enamel organ (EO) including outer enamel epithelium (OEE), stellate reticulum (SR), stratum intermedium (SI), and inner enamel epithelium (IEE) [ 1 - 2 ]. In the early bell stage of tooth development, cervical loop of incisor tooth germs continues to proliferate and begin to turn downwards. Tooth development involves several interactions between epithelial-mesenchymal cells. The IEE, a component of the enamel organ, gives rise to ameloblasts. Dental papilla mesenchymal cells differentiate to odontoblasts [ 3 ]. 

The dentin extracellular matrix (DECM) is composed of various non-collagenous proteins (NCPs) that play a pivotal role in the conversion of predentin into dentin. Dentin sialophosphoprotein (DSPP), which stands as the most prevalent non-collagenous protein in the dentin matrix, is initially secreted into the matrix and swiftly cleaved into two distinct proteins including dentin phosphoprotein (DPP) and dentin sialoprotein (DSP) [ 4 ]. DSPP's expression extends to multiple tissues, including dentin, cementum, and bone. Furthermore, DSPP finds expression in ameloblasts, encompassing both presecretory and secretory ameloblasts [ 5 ]. Beyond its presence in these tissues, DSPP plays crucial roles in the development of craniofacial structures [ 6 ]. Notably, the interplay of epithelial- mesenchymal interactions hold paramount importance in tooth development, and DSPP is a significant contributor to these interactions [ 7 - 8 ]. 

The human DSPP gene mutation is associated with dentinogenesis imperfecta types II and III. Besides, DS-PP has been found in tissues such as periodontal tissues, salivary glands, bone, mammary gland, and kidney [ 9 ]. 

The present study aimed to investigate the DSPP expression profile during early human primary tooth development with a sufficient sample size. 

## Materials and Method

All procedures were in accordance with the ethical standards of the Hamadan University of Medical Sciences. The Ethics Committee approval number was IR.UMSHA.REC.1398.682. 

### Sample preparation

In this experimental study, first, written informed consents from parents of all aborted human fetuses were obtained. The samples were then collected from 13- to 23-week-old fetal cadavers after legal abortion. The crown-rump length (CRL) of each embryo was measured to determine the embryonic age [ 9 ]. A dilation and curettage procedure were performed for pregnancies under 13 weeks’ gestation. Besides, according to the religion rules, fetuses ranging from 13 to 23 weeks of age (in increments of 3 weeks) were selectively gathered for this study, with fetuses beyond 23 weeks excluded. Subsequently, the heads of each specimen were meticulously severed and immersed in a 37% formaldehyde solution (formalin) for duration of two days. All samples were decalcified with dilute nitric acid (5%) and embedded in paraffin. From each paraffin block, sections were cut for hematoxylin and eosin (H&E) and immunohistochemistry (IHC) staining according to the previous studies [ 10 - 11 ]. The samples were divided into three different age groups: <16 weeks, <19 weeks and ≥19 weeks. The bell stage begins in the 14th week of gestation in human incisor tooth germ. In addition, dentin formation begins in the cusp tip of both the central mandibular and maxillary incisors around 16th week of gestation. Moreover, the IEE cells undergo differentiation into ameloblasts at 18^th^ week of gestation [ 12 ]. 

### The Performance of IHC

Antibody used in the IHC assay was Anti-DSPP rabbit polyclonal antibody (1:170, ab216892; Abcam, UK). Omission of the primary was used as the negative control. The cytoplasmic and extracellular matrix staining was analyzed. The percentage of stained cells was calculated for each cell type in all samples of primary central incisors and primary first molars in both jaws [ 13 ]. 

### Data analysis

Data was analyzed by using SPSS (version 20.0; SPSS Inc. Chicago, IL). A two-way ANOVA test analyzed the effect of fetal age and types of teeth on the expression level of DSPP in odontoblasts, cervical loop, and distinct layers of EO. Tukey’s post-hoc test assessed the significance of differences between the groups. The *p* values ≤ 0.05 were considered as significant.

## Results

### Histologic analysis for the expression level of DSPP in various structures of human developing primary teeth

In this study, 25 samples were males and 8 were females. Table 1 shows the analysis of DSPP expression level in the EO and some other histological structures of human developing.

**Table 1:** Analysis of DSPP expression in various structures of human developing primary teeth

Source of Variation	*df*	Mean square	F	*P* Value
Fetal age (IEE)	2	39641.346	908.981	0.000
Types of teeth (IEE)	3	1780.593	40.829	0.000
Interaction (IEE)	6	477.357	10.946	0.000
Fetal age (SI)	2	60025.455	346.161	0.000
Types of teeth (SI)	3	1888.886	10.893	0.000
Interaction (SI)	6	358.974	2.070	0.062
Fetal age (SR)	2	11312.574	746.778	0.000
Types of teeth (SR)	3	597.367	39.434	0.000
Interaction (SR)	6	113.556	7.496	0.000
Fetal age (OEE)	2	62643.008	723.753	0.000
Types of teeth (OEE)	3	2634.032	30.433	0.000
Interaction (OEE)	6	65.837	0.761	0.602
Fetal age (odontoblasts)	2	68023.618	383.126	0.000
Types of teeth (odontoblasts)	3	3244.724	18.275	0.000
Interaction (odontoblasts)	6	235.048	1.324	0.252
Fetal age (cervical loop)	2	59700.124	370.285	0.000
Types of teeth (cervical loop)	3	1843.835	11.436	0.000
Interaction (cervical loop)	6	109.188	0.677	0.668

IEE: Inner enamel epithelium; SI: Stratum intermedium; SR: Stellate reticulum; OEE: Outer enamel epithelium

The post-hoc analysis indicated a statistically significant difference between the DSPP expression level in the IEE layer of mandibular first molar and maxillary central incisor (*p*< 0.001), between the DSPP expression level in the IEE layer of maxillary first molar and maxillary central incisor (*p*< 0.001). However, no significant difference was found in terms of the DSPP expression level in the IEE layer (*p*< 0.998). 

In addition, the post-hoc test found a significant difference between the mandibular first molar and maxillary central incisor (*p*< 0.001), as well as the maxillary first molar and maxillary central incisor (*p*< 0.007) in terms of the DSPP expression level in the SI layer. Nevertheless, no significant difference was indicated between the mandibular central incisor and maxillary central incisor regarding the DSPP expression level in the SI layer (*p*< 0.998). 

In the SR layer, there was a significant difference between the mandibular first molar and maxillary central incisor (*p*< 0.001), as well as the maxillary first molar and maxillary central incisor (*p*< 0.001) regarding the DSPP expression level. However, no significant difference was observed between mandibular central incisor and maxillary central incisor (*p*< 0.153) in terms of the DSPP expression level.

Likewise, in the OEE layer, a significant difference in the DSPP expression level was found between the mandibular first molar and maxillary central incisor (*p*< 0.001), as well as in the maxillary first molar and maxillary central incisor (*p*<0.001). However, no significant difference was detected between the mandibular central incisor and maxillary central incisor (*p*< 0.567) regarding the DSPP expression level in the OEE layer.

The post-hoc test also found a significant difference between the odontoblasts of mandibular first molar and maxillary central incisor (*p*< 0.001) as well as the odontoblasts of maxillary first molar and maxillary central incisor (*p*< 0.001) regarding the DSPP expression level. However, no significant difference was found between the mandibular central incisor and maxillary central incisor (*p*< 0.869) in terms of the DSPP expression level in the odontoblasts.

**Figure 1:**
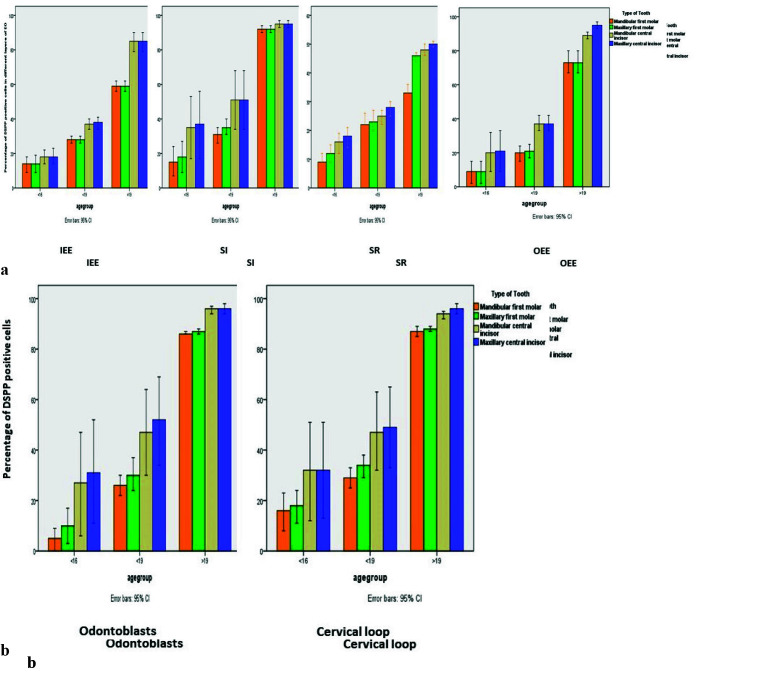
**a:** Histograms depicting the percentage of DSPP expression in different layers of EO, **b:** and in odontoblasts and cervical loop. IEE: inner enamel epithelium, SI: stratum intermedium, SR: stellate reticulum, OEE: outer enamel epithelium

**Figure 2:**
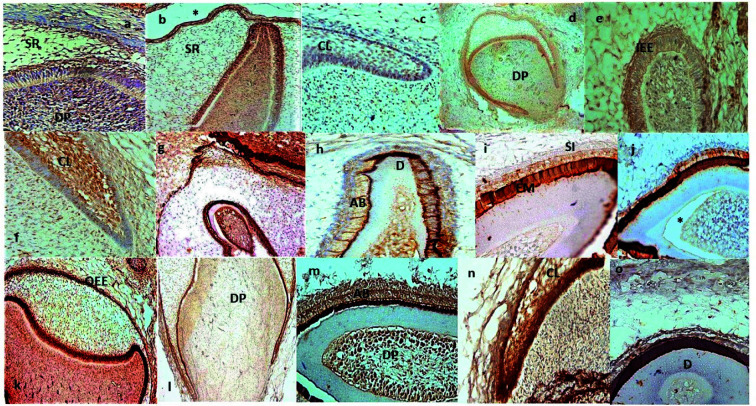
Expression pattern of DSPP in the human primary EO, odontoblasts and cervical loops in different fetal ages (a-o), (X100 or X400). **a:** 13g.w. (MaCI), **b:** 14g.w. (MCI), **c:** 15g.w. (MaCI), **d:** 16g.w. (MaFM), **e:** 16g.w. (MaCI), **f:** 17g.w. (MaCI), **g:** 18g.w. (MaCI), **h:** 18g.w. (MCI), **i:** 19g.w. (MCI), **j:** 20g.w. (MaCI), **k:** 20g.w. (MFM), **l:** 21g.w. (MaFM), **m:** 21.g.w.( MaCI), **n:** 22g.w. (MaCI), **o:** 23g.w. (MCI). Asterisks indicate artifactual detachment; g.w.: gestation week; AB: ameloblast; D: dentin; DP: dental papilla; EM: enamel matrix; IEE: inner enamel epithelium; OD: odontoblast; OEE: outer enamel epithelium; SI: stratum intermedium; SR: stellate reticulum; CL: cervical loop; MCI: maxillary central incisor; MaCI: mandibular central incisor; MFM: maxillary first molar; MaFM: mandibular first molar

Furthermore, a statistically significant difference in the DSPP expression level was demonstrated in the cervical loop between the mandibular first molar and maxillary central incisor (*p*< 0.001), as well as in the maxillary first molar and maxillary central incisor (*p*< 0.001). However, there was no significant difference between the mandibular central incisor and maxillary central incisor (*p*< 0.958) regarding the DSPP expression level in the cervical loop (Figure 1a, b) and (Figure 2a-o).

## Discussion

**Figure 3:**
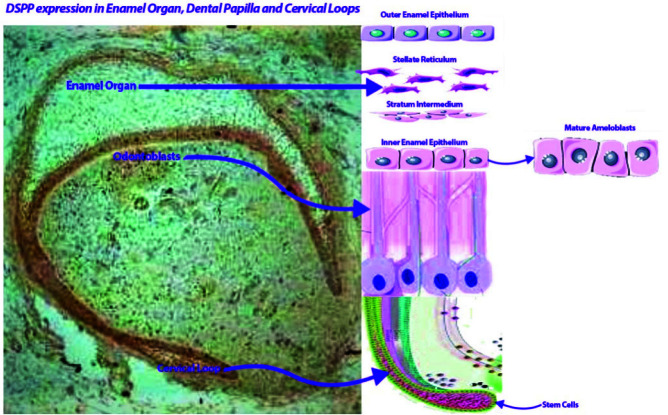
Graphical abstract

In this research, human developing teeth served as the primary subject for examining the expression level of DSPP across various dental structures. Commencing from the 13th gestational week, a notable immunepositive presence of DSPP was observed in specific dental pulp mesenchymal cells, which are believed to encompass stem cells and potential future odontoblasts. These DSPP-positive cells were particularly concentrated adjacent to IEE layer, which eventually gives rise to ameloblasts. Additionally, DSPP expression was detected in cells within the SR and OEE layers (as depicted in Figure 2-3). The mature odontoblasts of developing teeth also exhibited DSPP expression. A prior animal study demonstrated DSPP's role in driving stem cell differentiation into odontoblasts [ 14 ]. Notably, another animal study has demonstrated the presence of DSP in pre-ameloblasts and the SI layer of the developing mandibular first molar [ 5 ]. It has been suggested that DSPP may contribute to the differentiation of ameloblast as well [ 15 ]. However, the other studies have shown that DSPP is transiently expressed in early ameloblasts [ 5 , 14 ]. These results agree with the results that have been previously established about the DSPP expression in dental pulp cells, odontoblasts, and ameloblasts [ 5 , 14 , 16 - 19 ]. Furthermore, it was observed that DSPP expression increased across all cell types, as gestational weeks progressed (see Figure 1a, b). The consistently strong immunopositivity of DSPP across various cell types suggests its potential involvement in maintaining the tooth microenvironment, driving odontoblast and ameloblast differentiation, and contributing to dentin and enamel formation.

Furthermore, the expression of DSPP in pre-secretory and secretory ameloblasts and odontoblasts in areas subjacent to the dentino-enamel junction (DEJ) lends credence to the concept that DSPP may have a great impact on the formation of this interface [ 15 ]. In the present study, strong positive immunoreaction with DSPP antibody was found in ameloblasts and enamel matrix of incisors. This finding may prove the role of DSPP in enamel as well as DEJ formation.

Expression of DSPP in cervical loops in the current study and an increase in the expression level with the progression of gestational weeks may confirm the results of a previous study showing that stem cells reside in the cervical loops [ 8 ].

## Conclusion

The present investigation provides a detailed report on the expression of DSPP during human primary tooth development. There were some associations between the DSPP expression and the human primary teeth development. Based on this study, an increase in the expression level of DSPP with the progression of gestational weeks may confirm the reciprocal interactions between the EO and underlying mesenchymal cells (dental pulp stem cells) as well as contribution of DSPP in tooth development. Further investigation with larger sample size is required to study the role of DSPP in human primary tooth development. 

## References

[1] Liu X, Xie F, Lai G, Wang J ( 2020). Roles of heterogeneous nuclear ribonucleoprotein L in enamel organ development and the differentiation of ameloblasts. Arch Oral Biol.

[2] Irani S, Foroughi F ( 2017). Histologic Variants of Calcifying Odontogenic Cyst: A Study of 52 Cases. J Contemp Dent Pract.

[3] Imhof T, Rosenblatt K, Pryymachuk G, Weiland D, Noetzel N, Deschner J, et al ( 2020). Epithelial loss of mitochondrial oxidative phosphorylation leads to disturbed enamel and impaired dentin matrix formation in postnatal developed mouse incisor. Sci Rep.

[4] Wan C, Yuan G, Luo D, Zhang L, Lin H, Liu H, et al ( 2016). The Dentin Sialoprotein (DSP) Domain Regulates Dental Mesenchymal Cell Differentiation through a Novel Surface Receptor. Sci Rep.

[5] Park SJ, Lee HK, Seo YM, Son C, Bae HS, Park JC ( 2018). Dentin sialophosphoprotein expression in enamel is regulated by Copine-7, a preameloblast-derived factor. Arch Oral Biol.

[6] Chen Y, Zhang Y, Ramachandran A, George A ( 2016). DSPP Is Essential for Normal Development of the Dental-Craniofacial Complex. J Dent Res.

[7] Lim D, Wu KC, Lee A, Saunders TL, Ritchie HH ( 2021). DSPP dosage affects tooth development and dentin mineralization. PLoS One.

[8] Irani S, Alimohammadi S, Keyhan Shookoh S ( 2023). The role of amelogenin protein in the development of human primary teeth. Iran J Neonatol.

[9] Wyk LV, Smith J ( 2016). Postnatal foot length to determine gestational age: a pilot study. J Trop Pediatr.

[10] Irani S, Jafari B ( 2018). Expression of vimentin and CD44 in mucoepidermoid carcinoma: A role in tumor growth. Indian J Dent Res.

[11] Irani S, Dehghan A ( 2017). Expression of vascular endothelial-cadherin in mucoepidermoid carcinoma: Role in cancer development. J Int Soc Prev Community Dent.

[12] Hu X, Xu S, Lin C, Zhang L, Chen Y, Zhang Y ( 2014). Precise chronology of differentiation of developing human primary dentition. Histochem Cell Biol.

[13] Jensen EC ( 2013). Quantitative analysis of histological staining and fluorescence using Image. J Anat Rec.

[14] Lim D, Wu KC, Lee A, Saunders TL ( 2021). DSPP dosage affects tooth development and dentin mineralization. PLoS One.

[15] Verdelis K, Szabo-Rogers HL, Xu Y, Chong R, Kang R, Cusack BJ, et al ( 2016). Accelerated enamel mineralization in Dspp mutant mice. Matrix Biol.

[16] Mata M, Peydró S, de Llano JJM, Sancho-Tello M, Carda C ( 2022). Human dental pulp stem cells differentiate into cementoid-like-secreting cells on decellularized teeth scaffolds. Int J Mol Sci.

[17] Liu M, Li W, Xia X, Wang F, MacDougall M, Chen S ( 2021). Dentine sialophosphoprotein signal in dentineogenesis and dentine regeneration. Eur Cells Mater.

[18] Hao J, Ramachandran A, George A ( 2009). Temporal and spatial localization of the dentin matrix proteins during dentin biomineralization. J Histochem Cytochem.

[19] Liang T, Xu Q, Zhang H, Wang S, Diekwisch TGH, Qin C, et al ( 2021). Enamel defects associated with dentin sialophos-phoprotein mutation in mice. Front Physiol.

